# Zinc Ions Modulate YY1 Activity: Relevance in Carcinogenesis

**DOI:** 10.3390/cancers15174338

**Published:** 2023-08-30

**Authors:** Małgorzata Figiel, Adam Kazimierz Górka, Andrzej Górecki

**Affiliations:** Faculty of Biochemistry, Biophysics and Biotechnology, Department of Physical Biochemistry, Jagiellonian University, Gronostajowa 7, 30-387 Kraków, Poland; m.figiel@uj.edu.pl (M.F.); adam.kazimierz.gorka@gmail.com (A.K.G.)

**Keywords:** Yin Yang 1, zinc binding, dimerization, intrinsically disordered protein, protein–protein interaction

## Abstract

**Simple Summary:**

YY1 is a protein regulator of gene expression that has been shown to be involved in the progression of numerous cancers. We recently described a previously unrecognized effect of zinc ions on YY1’s structure and activity. The cellular zinc level is tightly regulated, but at the same time, it differs in normal and malignant cells, providing a context that could explain YY1 dysfunction. This review explores possible mechanisms through which YY1’s interaction with zinc ions might affect its activity, resulting in abnormal gene expression pattern.

**Abstract:**

YY1 is widely recognized as an intrinsically disordered transcription factor that plays a role in development of many cancers. In most cases, its overexpression is correlated with tumor progression and unfavorable patient outcomes. Our latest research focusing on the role of zinc ions in modulating YY1’s interaction with DNA demonstrated that zinc enhances the protein’s multimeric state and affinity to its operator. In light of these findings, changes in protein concentration appear to be just one element relevant to modulating YY1-dependent processes. Thus, alterations in zinc ion concentration can directly and specifically impact the regulation of gene expression by YY1, in line with reports indicating a correlation between zinc ion levels and advancement of certain tumors. This review concentrates on other potential consequences of YY1 interaction with zinc ions that may act by altering charge distribution, conformational state distribution, or oligomerization to influence its interactions with molecular partners that can disrupt gene expression patterns.

## 1. Introduction

Yin Yang 1 (YY1) is a pleiotropic regulator of numerous cellular processes. It binds a specific DNA motif that occurs in numerous gene regulatory elements, and thus regulates transcription of many genes, mostly involved in cell proliferation, differentiation, or apoptosis [1,2]. YY1 can either activate or repress gene expression depending on the molecular context. Its mechanisms of action include enhancer–promoter looping, recruitment of chromatin modulators, and direct interaction with regulatory proteins, i.e., p53, Rb1, or EZH2 [1,3,4,5,6]. YY1 malfunction causes serious consequences: YY1^−/−^ genotype causes peri-implantational lethality in mice [7] and YY1^+/−^ results in neurodevelopmental disorder in humans [8].

We recently found that zinc ions influence YY1 structure and activity [9]. Yin Yang 1 has long been known to bind four zinc atoms within zinc finger motifs of its DNA-binding domain [10]. Several (3–5) additional zinc ions can be bound by the N-terminal regulatory domain of YY1 [9]. This part of the protein is disordered in the apo form [11] but becomes more ordered upon binding Zn^2+^ [9]. The increase in order of an intrinsically disordered protein (IDP) is commonly attributed to alterations in its conformational dynamics. The binding of zinc ions presumably restricts the conformational flexibility of YY1, leading to a narrower distribution of available conformations. This is an expected result of the formation of coordination bonds around the ions which, in turn, stabilizes certain secondary structures. Furthermore, the presence of zinc ions facilitates YY1 dimerization through interactions involving its N-terminal domain. Both the conformational change and dimerization are specific to zinc ions, as no such effects were observed in the presence of different transition metal ions. Natural alterations in zinc ion concentration within cells can be part of a signaling pathway leading to modulation of biological activity of YY1.

## 2. Zinc Homeostasis

Cellular zinc levels are controlled by numerous proteins, suggesting that cellular zinc constitutes an important regulatory element. The concentration of zinc ions is significantly higher outside a living cell than inside. Due to the inability of zinc ions to spontaneously traverse the lipid bilayer, specialized transmembrane zinc-transporting proteins are engaged in their transportation. Two families of proteins with opposing effects on zinc ions are the ZIP proteins (Zrt-, Irt-like Proteins), facilitating transport of these ions into the cell, and the ZnT proteins (Zinc Transporters), actively pumping them out. The fine balance of these two protein families maintains zinc ion homeostasis, characterized by the maintenance of constant intracellular concentration. In humans, ten genes encoding ZnT and fourteen genes encoding ZIP have been identified that are differentially expressed depending on the cell type to support tissue specificity and a range of responses to external stimuli. Consequently, the intracellular zinc ion concentration is strongly dependent on the cell type. The presence of these proteins in the internal membranes of cells further contributes to differences in zinc ion concentrations within cellular compartments (Table 1). 

Additionally, cytoplasmic proteins and small molecular compounds that can chelate zinc ions (such as metallothioneins, citrate, histidine, cysteine, or glutathione) buffer its availability. This process is also precisely regulated. For example, an increased level of zinc ions increases the nuclear localization and DNA-binding activity of metal regulatory transcription factor (MTF-1), which activates expression of metallothioneins that decrease the accessible pool of zinc ions. 

The zinc-regulating proteins, and thus cellular zinc levels, are also differentially expressed and post-translationally modified in various tissues and conditions. Zinc levels are increased in cancers of breast, lung, nasopharynx, and intestine [92]. In breast cancer, overexpression of zinc importers ZIP6, 7, and 10 correlates with malignancy [38,47,93]. Loss of the ZnT2 transporter is frequently observed in malignant breast cancer, limiting storage of zinc in intracellular vesicles [63]. On the other hand, the cellular zinc level decreases in cancers of prostate, pancreas, liver, gallbladder, and cervix [92]. The highest drop in zinc level is observed in prostate cancer, where it correlates with malignancy and is considered a prognostic marker [94]. A decreased zinc level in prostate cancer cells is probably caused by downregulation of ZIP1, 2, and 3 and the ZnT4 transporter [14,15,65]. A decrease in zinc level and downregulation of ZIP3 are also considered to occur early during the progression of pancreatic cancer [21]. More examples of changes in expression of zinc-regulating proteins are presented in Table 1. Restoring the physiological level of zinc has been shown to decrease the proliferation of prostate cancer cells both cultured in vitro and in xenograft models. The underlying mechanisms included activation of p53 and p21 through the PTEN/AKT/MDM2/p53 and AKT/p21 pathways; IGF-1 signaling; downregulation of androgen receptor (AR); and induction of mitochondrial-mediated apoptosis (reviewed in [95]). Zinc homeostasis is considered a potential therapeutic target in treatment of various cancers [17,30,74].

## 3. Cellular Zinc Level—What Is the True Meaning?

The level of free zinc ions in the cytoplasm was first estimated to be sub-nanomolar based on the zinc concentration dependence of various zinc-binding proteins, including the regulator MTF-1 [96], which is activated by zinc, or phosphoglucomutase, which is inhibited by zinc [97]. Direct measurements were later performed in living cells with the use of fluorescent zinc chelators including Zinquin, Mag Fura-5, or FluoZin-3. These confirmed the initial sub-nanomolar limit and allowed quantification of the differences among cell types and conditions. Generally, zinc levels fell within the high picomolar to low nanomolar range [98,99,100]. It is worth noting that zinc-binding sensors can affect the intracellular zinc equilibrium, especially if they accumulate in the cytoplasm [101]. Some of the zinc probes, e.g., Zinquin, can also detect protein-bound zinc [102]. Thus, the probes are best used for comparisons and not for direct concentration measurement [103]. 

In contrast, the total cellular zinc levels vary over the sub-millimolar range in different tissues and conditions [101]. Cellular zinc is regulated through different fluxes into the cytoplasm, as well as through sequestering zinc ions by chelators, binding proteins, and organelles. In addition, the cell nucleus, mitochondria, endoplasmic reticulum, Golgi, and endosomes can act as zinc reservoirs, sequestering part of the ions but also able to release them upon a trigger. The zinc ions present in the cytoplasm or nucleus might also be tightly bound by proteins. This pool contains the “structural” and “catalytic” zinc ions. The remaining ions are chelated by other proteins, mainly metallothioneins or small molecule chelators such as citrate, histidine, cysteine, or glutathione [104]. Their affinity is low to moderate, so the ions are still accessible for other compounds. The concentration of such exchangeable zinc has been estimated to be ∼5–100 μM by Costello and Franklin [105]. The authors further augment their calculations with the observation that the estimation lays in the range of K_m_ values for numerous zinc transporters and enzymes. Alternatively, the seemingly too low metal affinities of various proteins can be explained by tight cellular regulation of zinc availability, achieved through dynamic redistribution between subcellular pools [106]. Such restricted relative availability is obviously difficult to mimic in vitro. 

Thus, the labile zinc fraction in cells cannot be considered a universal and absolute value. It depends on the particular chelator under consideration, specifically relying on its binding affinity (K_d_) for zinc ions relative to the K_d_ of other compounds buffering the ions. The abundance of the chelator will also be crucial. Consequently, there is a continuum of concentrations of available zinc for various molecules characterized by their specific affinities. Naturally, the available zinc fraction will be greater for compounds exhibiting higher affinity towards zinc ions. For instance, zinc availability is higher for metallothioneins (K_d_ = 10^−14^–10^−11^) than for MTF-1 (K_d_ = 10^−11^–10^−6^), enabling their balanced functioning as a regulatory system [107,108]. 

## 4. Zinc Ions Affect the Structure and Function of Yin Yang 1

YY1 dimerization and oligomerization has been observed in numerous reports, in vitro [6,109,110,111,112,113] and in living cells [110]. It has also been shown that this dimerization has physiological significance. When ectopically expressed in breast cancer cells, wild-type YY1 promotes cell proliferation significantly less than mutants with decreased dimerization propensity [113]. 

YY1 regions engaged in zinc binding and zinc-triggered dimerization include the acidic region (aa 30–60) and the histidine cluster (aa 65–80), consistent with analyses showing that histidine, aspartate, and glutamate residues are most frequently engaged in zinc binding within protein oligomer interfaces [114]. On the contrary, Qiao and colleagues mapped the YY1’s dimerization interface to aa 201–226 [113]. The discrepancy may result from the lack of zinc ions in their surface plasmon resonance (SPR) experiments. Both reports agree, however, that the N-terminal part of YY1 is engaged in dimerization, and thus the resulting dimer contains two C-terminal DNA-binding domains. This affects both DNA binding strength and the recognized sites, which preferentially contain a double YY1 core motif [9]. Similar zinc dependence of DNA binding was observed for MTF-1, with a single core motif bound in low-zinc conditions versus a double motif preferentially bound in 100 μM zinc [115].

Since the activity of YY1 is dependent on the concentration of zinc ions, one might hypothesize that this protein could serve as a regulator in maintaining the homeostasis of these ions by controlling the expression of the other zinc-regulating proteins. Such feedback could lead to compensatory changes in zinc ion levels, ultimately minimizing alterations in YY1 activity. However, it has not been observed that YY1 is involved in the regulation of any of those proteins. Therefore, its role as an effector of changes in zinc ion concentration is presently unclear but may include other biological processes in which YY1 is engaged.

## 5. YY1—A Protein of Numerous Activities and Binding Partners

By protein activity, we understand its ability to efficiently perform its functions. This concept is particularly well-defined in the case of enzymes, where activity can be quantitatively determined by examining the rate of the enzymatic reaction catalyzed by the protein. For transcription-regulating proteins, activity refers to their ability to modify the level of the resulting transcript. An activator will have higher activity (will promote the transcription process to a greater extent) the more its presence increases the level of the transcript, while a repressor will have higher activity (will inhibit the transcription process to a greater extent) the more it decreases the level of the transcript. However, gene expression regulation in eukaryotes typically does not rely solely on single proteins but rather on protein complexes. Various complexes are recruited to control the expression of particular genes, depending on specific binding sites within the promoter/operator region that can be recognized by individual transcription factors. In this case, the mutual affinity between different transcription factors is as important as the affinities of individual proteins for DNA. These interactions are interdependent, leading to the emergence of complex cooperativity and ultimately resulting in highly intricate and multifactorial gene expression regulation. This is particularly evident for the YY1 protein, which, depending on its partners and their recruitment by specific promoters, can stimulate gene expression as an activator or inhibit it as a repressor [2]. 

The YY1 protein with intrinsically disordered regions (IDRs) is particularly suited to mediating interactions with multiple proteins and thus carrying out its function in a partner-dependent manner within specific complexes. Modification of the structural properties of the IDR region can affect partner affinities, either promoting or inhibiting formation of complexes that control gene expression. Modification of the structure can have various, including opposing, effects on the activity of these complexes, either increasing or decreasing transcriptional activity. 

YY1 has been shown to interact with over two hundred different protein partners. Binding of zinc ions by YY1 may therefore lead to modulation of the interaction with at least some of these proteins. In order to map YY1 protein interactions with molecular partners, we analyzed the available literature data from coIP, two hybrid, and other experiments. The summary is presented in Table 2. Over two hundred different protein partners of YY1 were confirmed in these experiments. For 49 of the partners, the region of YY1 involved in the interaction was determined through studies on its deletion mutants. 

Studies summarized in Table 2 employed different methodological approaches, characterized by various limitations. Co-immunoprecipitation, bimolecular fluorescence complementation (BiFC), and two-hybrid assay have the advantage of a native, cellular context. Nonetheless, overexpression of the target proteins may result in artifacts, especially in the case of transient expression. Complexes of the overexpressed target protein might be overrepresented compared to their natural abundance, facilitating detection of labile and physiologically irrelevant complexes. On the other hand, in vitro assays such as co-purification or SPR lack all the components of a complex cellular environment but can provide increased resolution when protein fragments are analyzed. Co-purification and co-immunoprecipitation with mass spectrometry detection allow the discovery of binding partners with no prior assumptions. However, they can also detect indirect binding. Co-purification, SPR, two-hybrid screening, and BiFC require at least one of the interactors to be fused to another peptide: a tag, DNA-binding or activator domain, or fluorescent protein. This can affect the interaction under investigation, but, at the same time, ensures higher specificity of detection. Co-immunoprecipitation, although tag-free, should be interpreted with caution in the case of human YY1, as most of the available antibodies also recognize its paralog, YY2 [116,117]. The greater the variation in the methods employed to evaluate YY1 binding to its putative partners, the higher the credibility of the interactions identified. Because of the aforementioned limitations, results of the protein–protein interaction studies should be treated as qualitative, and their physiological meaning should be confirmed with different methods.

## 6. Regions of YY1 Molecule Responsible for Interactions with Its Molecular Partners

**Figure 1:**
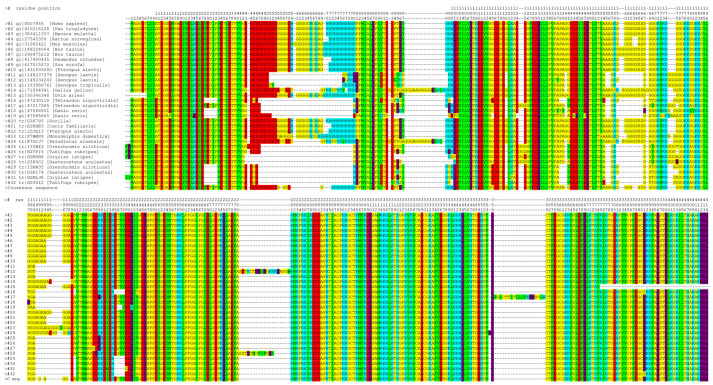
YY1 homology multiple sequence alignment shows its high evolutionary conservation. The alignment was performed using TranslatorX [120] and MAFFT [121]. Conserved residues are highlighted, using a modified Lesk color scheme (green—hydrophobic residues; yellow—small nonpolar; red—acidic; blue—basic; magenta—polar). Differences between the homologous sequences occur mainly outside the areas proposed in the further part of the work as molecular recognition features (MoRFs).

The inherent flexibility of IDPs enables them to undergo conformational adjustments as needed to establish interaction interfaces. This conformational adaptability facilitates interactions with many partners. The interactions can still be specific, despite their dynamic nature, because the conformation that a given polypeptide fragment can adopt depends on its sequence, and only some of the structures adopted by an IDP can be stabilized by its partner. The regions of IDPs that can undergo structurization are referred to as MoRFs, i.e., molecular recognition features. Such elements can transiently adopt secondary structures typical for globular proteins, i.e., β strands or α helices that can support molecular recognition of a suitable binding partner. The formation of a complex stabilizes the regular structure of the MoRF through a “folding upon binding” mechanism [118]. Determining the preferences of a peptide chain to adopt specific secondary structures may therefore be helpful in identifying regions suspected of being MoRFs, and thus may be helpful in isolating protein regions that interact with molecular partners partially independently from other regions. Segments of proteins exhibiting MoRF properties should be highly conserved evolutionarily [119]; therefore, to aid in their identification, multiple alignments of various YY1 sequences from diverse organisms were performed (Figure 1). 

**Figure 2:**
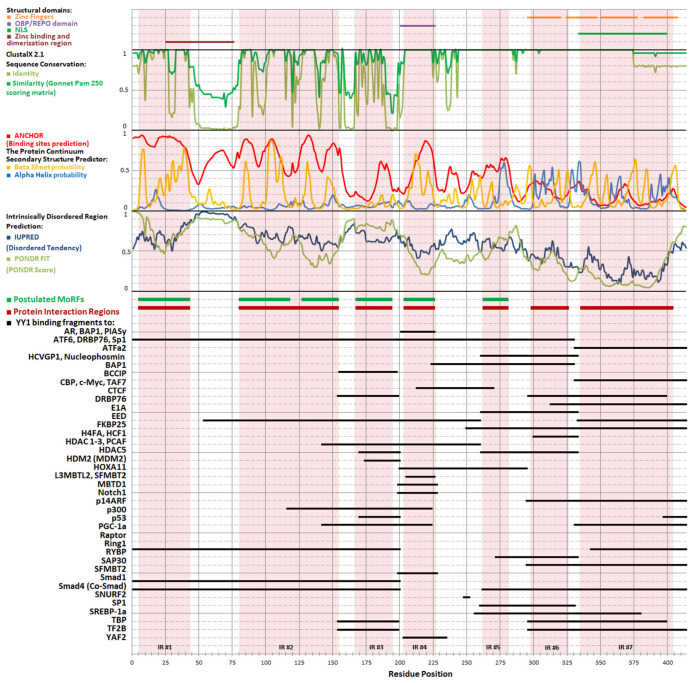
Seven interaction regions (IRs) proposed for YY1 based on bioinformatic analyses and protein–protein interaction experiments as described in the text. From top: structural domains; sequence conservations (1 denoting most conserved and 0 least conserved); MoRFs and secondary structure predictions of YY1 (1 denoting highest probability and 0 lowest probability of regular structure arrangement); IDR predictions (1 denoting highest and 0 lowest disorder tendency); postulated Interaction Regions and fragments of YY1 interacting with partners (detailed data on interactions with partner proteins are provided in Table 2). The proposed interaction regions are characterized by high evolutionary conservation, high index of binding site prediction, and high tendency to form secondary structures, and have been experimentally shown to participate in interactions with molecular partners. The first five postulated regions are located in the area considered as intrinsically disordered and coincide with the postulated MoRFs, and the remaining two are elements with a stable spatial structure: first zinc finger and the remaining three zinc fingers.

Based on this alignment of YY1 homolog sequences, several bioinformatic predictions have been made: Intrinsically Disordered Region with PONDR FIT [122], IUPRED [123], and secondary structures and MoRFs with ANCHOR program [124]. The obtained results correlate well with sequence conservation and experimentally determined protein–protein interactions regions (Figure 2), as the highly conserved regions of YY1 show a greater tendency to form regular structures, mainly β strands, than the regions that contain indels or point mutations. This joined analysis allowed us to distinguish seven regions within YY1 for protein–protein interactions that are predicted to temporarily form regular structure motifs, called here Interaction Regions (IR1-7). 

IR1, located at the N-terminus of YY1, includes residues 6–42 (numbers refer to the human protein), and is marked by high content of order-promoting hydrophobic residues, intertwined with acidic residues. Highly conserved sequences 16–28 and 33–42 are predicted to form α-helical and β-strand structures, respectively. IR2 (residues 81–153) also contains intertwined hydrophobic and acidic residues, separated by regions with a higher frequency of mutations. E1A and p53 are known to interact with this region of YY1 [5,125]. IR3 is formed by residues 168–193, which are mainly polar, with a high content of glycine, serine, and alanine residues, but also several highly conserved lysines. Such a composition may be responsible for IR3’s intrinsic disorder and promotes numerous interactions and posttranslational modifications. IR3 binds to histone acetylases p300, CBP, and PCAF; deacetylase HDAC 1-3; chromatin modifier CTCF; general transcription factors TAFII55, TBP, and TFIIB; and regulatory proteins c-Myc, p53, and nucleophosmin [5,125,126,127,128,129,130]. IR4 (204–225) is unique among the IRs with a known spatial structure, resolved for a cocrystal with malignant brain tumor domain-containing protein 1 (MBTD1) [131]. The crystal structure includes two β strands, in line with the bioinformatic predictions. Zhang et al referred to the region of IR4 as the oncoprotein binding domain (OPB) [132], while Wilkinson et al. proposed the name Recruitment of Polycomb (REPO) domain, highlighting its role in the interaction with PcG group proteins [133]. Other protein interactors of IR4 include the MBTD1 homolog L3MBTL2 and SFMBT2, as well as AKT, EZH2, MDM2, HOXA11, and Raptor [113,131,134,135]. IR5 (263–280) shows helix propensity and was shown to be involved in binding to ATFa2 and ATF6 proteins [136]. We propose to separate the zinc finger domain of YY1 into two distinct regions: IR6 spanning residues 299–325 (i.e., zinc finger 1), and IR7, which includes residues 336–403 (i.e., zinc fingers 2–4). The first zinc finger and the linker between the first and second finger differ from the canonical sequence [2], and are dispensable for DNA binding [10,137]. The remaining fingers and linkers correspond more closely to the consensus and are required for DNA binding. IR6 has a predicted higher tendency for disorder than IR7 and some distinct protein partners. CP2, SP1, FKBP25, and RYBP interact solely with IR6 [138,139,140,141], while AR, BAP1, CTCF, PGC1α, and PIASγ interact solely with IR7 [128,134,142,143,144]. 

Several observations can be made regarding the contribution of IRs to YY1’s protein–protein interactions. First, interactions with some partners engage more than one IR, e.g., many transcriptional activators recognize jointly IR1 and 2. Interactions with transcriptional repressors often engage IR3, 6, and 7. Second, some proteins interact independently with two distal IRs, e.g., CBP, E1A, HDAC1-3, c-Myc, p300, p53, PCAF, Ring1, TBP, or TF2B. Finally, some IRs are self-sufficient for the protein binding, e.g., PcG proteins interact only with IR6.

The interactions of many proteins with the same IR suggest a possible mutually competitive nature. On the other hand, the interaction of a single partner with different IRs can give rise to synergy, resulting in stronger and more specific binding. This creates opportunities to regulate transcription.

**Table 2:** Interaction partners of YY1.

Partner Protein	Partner Fragment	YY1 Fragment	Additional Partner	Method	Reference
AMRP (α-2-macroglobulin receptor-associated protein)	FL	1–414		CP	[145]
AAMDC (Mth938 domain-containing protein)	FL	1–414		CP	[145]
AGO2 (Argonaute 2)	FL	1–414		coIP	[146]
AKT (PKB)	1–108	201–226		CP, coIP	[113,132]
ALOXE3	FL	1–414		2H	[147]
ALR	FL	1–414		CP, coIP	[148]
AP2	166–437	1–330		CP, coIP	[149,150]
AR (androgen receptor)	556–919	331–414		CP	[142]
ARB1 (β-arrestin)	FL	1–414		coIP	[151]
ATF2	FL	1–414		CP	[136]
ATF6	273–373	261–333		CP	[152,153]
ATFa1	FL	1–414		CP	[136]
ATFa2	334–399	224–330		CP	[136]
ATFa3	FL	1–414		CP	[136]
ATXN2L	FL	1–414		CP	[145]
Aurora A (AURKA)	FL	1–414		CP	[154]
BAP1	642–686	331–414	HCF-1	CP, coIP	[143]
BAX	FL	1–414		CP	[155]
BCCIP	1–258	213–270		CP, coIP	[156]
BCL6	FL	1–414		CP	[157]
BMI1	FL	1–414		CP	[158,159,160]
BRD1	FL	1–414		CP	[161]
BRD2	FL	1–414		CP	[161]
BRD4	FL	1–414		CP	[162]
CAND-1	FL	1–414		coIP	[163]
CAPB	FL	1–414		CP	[164]
CBP	451–721	154–199; 296–399		CP, coIP	[127,165]
CBX4	FL	1–414		CP	[145]
CCNT1	FL	1–414		CP	[145]
CCNT2	FL	1–414		CP	[145]
CDK9	FL	1–414		CP	[145]
C/EBP β	FL	1–414		CP	[166]
CEP76	FL	1–414		2H	[147]
CHD8	FL	1–414		CP	[164]
CIC	FL	1–414		CP	[167]
CKIδ	FL	1–414		CP	[145]
cortactin	FL	1–414		CP	[145]
CP2	308–368	294–320	HDAC1	CP	[138,168,169]
CPSF1	FL	1–414		CP	[145]
CPSF6	FL	1–414		CP	[145]
CPSF7	FL	1–414		CP	[145]
CREB (ATF)	FL	282–414		CP, 2H	[136,170]
CRKL	FL	1–414		2H	[171]
CTCF	1–583	313–414		CP, coIP	[128]
CUL3 (cullin 3)	FL	1–414		coIP	[172]
Cyclophilin A	FL	1–414		2H	[173]
CYSRT1	FL	1–414		2H	[147]
DCAF13	FL	1–414		CP	[145]
DDX3X	FL	1–414		coIP	[174]
DDX5	FL	1–414		coIP	[174]
DDX6	FL	1–414		CP	[145]
DDX42	FL	1–414		CP	[145]
DDX56	FL	1–414		CP	[145]
Dot1L	FL	1–414		coIP	[175]
DNAPK	FL	1–414		CP	[112]
DNMT3L	FL	1–414		PA	[176]
DRBP76	FL	261–333		CP, coIP	[177]
E1A	15–35; 140–188	54–260; 332–414	p300	CP, CS, FWB	[125,136,178,179,180,181]
EED	502–535	250–414		2H, CP, coIP	[4]
EIF5A	FL	1–414		CP	[145]
ESM1	FL	1–414		2H	[147]
EVI1	FL	1–414		CP	[182]
EZH2	493–519	201–226		SPR, CP, coIP	[4,113,183,184]
FAM76A	FL	1–414		CP	[145]
FAM67B	FL	1–414		CP	[145]
FAM98A	FL	1–414		CP	[145]
FBW7	FL	1–414		coIP	[185]
FHL2	FL	1–414		2H	[147]
FIP1	FL	1–414		CP	[145]
FKBP12	FL	1–414		2H	[173]
FKBP25	1–90	300–333		CP, coIP	[139]
c-Fos	FL	1–414		coIP	[186]
FOX-B1	FL	1–414		CP	[187]
FOX-J2	FL	1–414		CP	[187]
FOX-L1	FL	1–414		CP	[187]
FOX-N1	FL	1–414		CP	[187]
GMCL1	FL	1–414		2H	[147]
GON4L	611–1364	1–414		CP, coIP	[188,189]
granulin (GRN)	FL	1–414		2H	[171]
H4FA	FL	142–260		CP	[190]
HCF-1	FL	142–260	BAP1	CP	[143]
HCVGP1 HCV core fusion protein	FL	1–414		CP, coIP	[130]
HDAC1	FL	170–200; 261–333		BA, CP, coIP	[126,191,192,193]
HDAC2 (RPD3)	FL	170–200; 261–333	DNA	BA, CP, coIP	[126,191,192,194,195,196,197]
HDAC3	373–428	170–200; 261–333	p300	BA, CP, coIP	[126,181,191,198,199]
HDAC3a	FL	170–200; 261–333		CP	[191]
HDAC4	FL	1–414		CP	[200,201,202]
HDAC5	FL	1–414		coIP	[203,204]
HEXIM1	FL	1–414		CP	[145]
HMGB1B	FL	1–414		2H	[205]
HOXA11	229–314	205–226	HDAC2	CP, coIP	[135]
HSPA4	FL	1–414		CP	[112]
IL-10	FL	1–414		2H	[147]
INO80 (KIAA1259)	273–521	1–414		CP, coIP	[112,145,164,206,207]
INO80B	FL	1–414		CP	[206]
INO80C	FL	1–414		CP	[164,206]
INO80D	FL	1–414		CP	[206]
INO80E	FL	1–414		CP	[145,206]
INO80F	FL	1–414		CP	[145,206]
INO80G (NFRκB)	FL	1–414		CP	[145,164,206]
INO80H (RUVBL1/TIP49A)	FL	1–414		CP, coIP	[112,145,164,206]
INO80J (RUVBL2/TIP49B)	FL	1–414		CP, coIP	[110,112,164,174,206]
INO80K (ACTL6A)	FL	1–414		CP, coIP	[112,164,206]
INO80M (ACTR5)	FL	1–414		CP	[112,155,164,206]
INO80N (ACTR8)	FL	1–414		CP, coIP	[112,145,164,174,206]
INO80Q (MCRS1)	FL	1–414		CP	[145,206]
INO80R (UCHL5)	FL	1–414		CP	[145,164,206]
ITFG-1	FL	1–414		CP	[208]
c-Jun	FL	1–414		coIP	[209]
JunB	FL	1–414		coIP	[210]
JunD	FL	1–414		coIP	[210]
KP1-3	FL	1–414		2H	[147]
KAP1-5	FL	1–414		2H	[147]
KAP2-3	FL	1–414		2H	[147]
KAP2-4	FL	1–414		2H	[147]
KAP4-2	FL	1–414		2H	[147]
KAP4-5	FL	1–414		2H	[147]
KAP5-6	FL	1–414		2H	[147]
KAP9-3	FL	1–414		2H	[147]
KAP9-8	FL	1–414		2H	[147]
KAP10-5	FL	1–414		2H	[147]
KAP10-8	FL	1–414		2H	[147]
KAP10-9	FL	1–414		2H	[147]
KAP12-2	FL	1–414		2H	[147]
KAP12-3	FL	1–414		2H	[147]
KAP17-1	FL	1–414		2H	[147]
Ki-67	FL	1–414		CP	[211]
Ku70	FL	1–414		coIP	[212]
Ku80	FL	1–414		coIP	[212]
L3MBTL2	170–625	199–228		SPR	[131]
LHX3	FL	1–414		2H	[147]
LHX4	FL	1–414		2H	[147]
LYAR	FL	1–414		CP	[145]
MAX	FL	1–414		coIP	[165]
MBTD1	130–566	199–228		SPR, CC	[131]
MDFI	FL	1–414		2H	[147]
MDM2 (HDM2)	150–290	200–295	p53	CP, coIP	[5,113]
MEPCE	FL	1–414		CP	[145]
MeCP2	202–255	1–414		CP, coIP	[213]
MED20	FL	1–414		2H	[147]
MEN1 (menin)	FL	1–414		coIP	[175]
METTL17	FL	1–414		CP	[145]
MFAP1	FL	1–414		CP	[145]
MLL5	FL	1–414		CP	[214]
MMTAG2	FL	1–414		CP	[145]
MSL2	FL	1–414		CP	[164]
MTA2	FL	1–414		CP	[215]
mTOR	FL	1–414	Raptor	coIP	[134]
c-Myc	262–439	154–199; 296–399		2H, CP	[129,216]
n-Myc	FL	1–414		coIP	[217]
NCAP (SARS-CoV-2)	FL	1–414		CP	[218]
NEDD4	FL	1–414		BA, coIP	[219]
NEDD4L	FL	1–414		BA, coIP	[219]
NFκB	FL	1–414		2H	[171]
NIRF	FL	1–414		coIP	[220]
Notch1	1821–2095	295–414		CP, coIP	[221]
NR1H2 (nuclear receptor 1H2)	FL	1–414		2H	[171]
NRF2	FL	1–414	DNA	coIP	[222]
NSRP1	FL	1–414		CP	[145]
Nucleophosmin	127–144	155–198		CP, coIP	[130]
NUDT21	FL	1–414		CP	[145]
NUFP2	FL	1–414		CP	[145]
p14ARF (CDKN2A, INK4)	FL	116–224		CP, coIP	[5]
p27 (CDKN1B, KIP1)	FL	1–414		CP	[223]
p53	290–393	142–224; 331–414		CP	[5,144,223,224,225]
p300	1572–2370	170–200; 397–414	HDAC3, c-Myc, Max	BA, 2H, CP, coIP	[125,126,170,181,199]
PARP1 (ADPRT)	337–573	1–414		BA, CP, coIP	[226,227,228]
PCAF	FL	170–200; 261–333		BA	[126]
PCGF2 (rnf110)	FL	1–414		CP	[160]
PGC-1α	400–797	350–380		CP, coIP	[134]
PIASγ	100–202	331–414		CP, coIP	[144]
PIRH2	FL	1–414		CP	[229]
PIPK	FL	1–414		CP	[145]
PKHF2	FL	1–414		2H	[147]
PLEKH4	FL	1–414		CP	[230]
POGZ	FL	1–414		CP	[164]
POP1	FL	1–414		CP	[145]
PPIL4	FL	1–414		CP	[145]
PPP1R10	FL	1–414		CP	[145]
PR38A	FL	1–414		CP	[145]
PR40A	FL	1–414		CP	[145]
PRMT1	FL	1–414		coIP	[153,177]
PRP4	FL	1–414		CP	[145]
PSP1	FL	1–414		CP	[145]
Raf-1	FL	1–414		2H	[171]
Raptor	FL	203–235		coIP	[134]
RB1	FL	1–414		coIP	[3,231]
RBM15B	FL	1–414		CP	[145]
RBM25	FL	1–414		CP	[145]
RCL1	FL	1–414		CP	[145]
Rel-B	FL	1–414		coIP	[232]
RhoGAP	FL	1–414		CP	[233]
RhoGEF	FL	1–414		CP	[233]
Ring1	FL	1–200; 343–414		CP	[160]
RNAP II (large subunit)	FL	1–414		CP	[170]
RNF2	FL	1–414		CP	[158,160]
RNF144	FL	1–414		CP	[234]
RPL23A	FL	1–414		CP	[145]
RPS19	FL	1–414		CP	[145]
RPSA	FL	1–414		CP	[145]
RYBP (YEAF1)	42–118; 207–227	272–333	GABPB1	CP, coIP, 2H	[141,235]
SAP30	129–220	295–414	HDAC1	CP, 2H	[192]
SART1	FL	1–414		CP	[145]
SF3A2	FL	1–414		2H	[147]
SF3B4	FL	1–414		CP	[145]
SFMBT2	44–447	199–228		SPR	[131]
SLC39A7	FL	1–414		2H	[171]
Smad1	12–136	1–200		CP, coIP	[236,237]
Smad2	10–176	1–414		CP, coIP	[236,238]
Smad3	10–136	1–414		CP, coIP	[236,238]
Smad4 (Co-Smad)	18–142	1–200		CP, coIP	[236,237]
Smad7	261–426	1–414		CP, coIP	[239]
SMURF2	FL	248–252		CP	[240]
SNIP1	FL	1–414		CP	[145]
SMARCAD1	FL	1–414		CP	[241]
Sp1	620–778	260–331	DNA with SP1 site	CP, coIP	[140,242,243,244,245]
Sp3	FL	1–414		coIP	[245]
Sp100	FL	1–414		CP	[145]
SPRTN	FL	1–414		CP	[246]
SPRY1	FL	1–414		2H	[171]
SREBP-1a	321–490	256–354		CP	[243]
SUZ-12	FL	1–414		CP	[158]
TACO1	FL	1–414		CP	[145]
TAF2	FL	1–414		CP	[145]
TAF7 (TAFII55)	1–117	154–199; 296–399		CP	[127,247]
Tat (HIV-1)	FL	1–414		CP	[248]
TBP	FL	154–199; 296–399		CP	[127,129,170]
TCF3	FL	1–414		CP	[206]
TESK1	FL	1–414		2H	[171]
TF2B	FL	154–199; 296–414		CP	[127,129,249]
TF2I	FL	1–414		CP	[145]
TOP1	FL	1–414		CP	[145]
TOX4	FL	1–414		CP	[145]
TRABID	FL	1–414		CP	[145]
TRF-1	FL	1–414		CP	[250]
TRF-2	FL	1–414		CP	[250]
TRIM42	FL	1–414		2H	[147]
TRIM67	FL	1–414		coIP	[251]
TRIP12	FL	1–414		coIP	[163]
TWIST-1	FL	1–414		CP	[225]
Ubiquitin B (UBB)	FL	1–414		coIP	[163]
Ubc9	FL	1–414		BA, CP, coIP	[144]
USP7	FL	1–414		CP	[252]
USP8	FL	1–414		coIP	[163]
VWC2	FL	1–414		2H	[147]
WDR82	FL	1–414		CP	[145]
WIZ	FL	1–414		CP	[164]
XAGE1B	FL	1–414		2H	[147]
XAGE1E	FL	1–414		2H	[147]
YAF2	FL	271–333		2H	[195]
YY1	65–80; 201–226	65–80; 201–226		SEC, BiFC, EM, SPR, CP	[6,9,110,111,113]
YY1AP	1–260; 475–608	1–414		2H, CP, coIP	[253]
YY2	FL	1–414		CP	[164]
ZHX1	FL	1–414		CP	[164]
ZNF85	FL	1–414		2H	[147]
ZNF232	FL	1–414		2H	[254]
ZNF644	FL	1–414		CP	[164]

The following designations are used: CP (co-purification); coIP (co-immunoprecipitation); CS (co-sedimentation); 2H (two-hybrid); SPR (surface plasmon resonance); BA (biological activity); FWB (far western blot) CC (co-crystallization); SEC (size exclusion chromatography), BiFC (bimolecular fluorescence complementation), EM (electron microscopy), PA (protein array) FL (full length).

## 7. YY1 Binding of Zinc Ions May Interfere with the Interactions of Molecular Partners

The flexibility of IDPs results from their deficit of hydrophobic residues and high uncompensated electrostatic charge. Thanks to these, IDPs can adopt conformations suitable for interaction with different molecular partners, and thus can bind to many more partners than ordered proteins [1]. The dynamic nature of the interaction results from the ability of the IDP to adopt conformations that favor binding. The binding of zinc ions changes both the charge distribution and the protein’s conformational freedom. The interaction depends on the fit between the spatial distribution of the metal ion orbitals [255] and the available structures of the polypeptide chain. Binding has specific nature thanks to the steric hindrance of the polypeptide chain and different distribution of the orbitals of particular metal ions. Such specific interaction is assumed to greatly decrease the flexibility of the protein, changing the binding interface and the interaction profile. 

Our NMR studies have indicated YY1 regions that undergo such changes upon zinc binding, particularly the acidic region centered around residue ~50. Subsequent NMR signal changes occur for residues 1–20, which also include scattered acidic residues, and the region around residue 110 including the histidine cluster (Figure 2). Thus, the binding of zinc ions is likely to primarily affect protein–protein interactions that involve IR1 and IR2. The previously described sequence order of the disturbances observed in the NMR spectrum, reflecting the preferences of zinc ions in the interaction with the N-terminus of YY1, suggests that increasing the concentration of zinc will firstly modulate the interactions with IR1 and then those with IR2. YY1 oligomerization could also hinder some of the interactions by competition with and modification of the binding interface. On the other hand, those IRs that are not engaged in zinc binding will be grouped together upon YY1 oligomerization. Several examples are provided below, where YY1 dimerization can explain the mechanism of its interactions with protein partners. The partners are known oncogenes in prostate tumorigenesis, a process accompanied by decreasing zinc level. 

### 7.1. EZH2 

Histone methyltransferase EZH2 functions within the Polycomb Repression Complex 2 to repress transcription of tumor suppressors [256]. Its overexpression correlates with the aggressiveness of prostate cancer. YY1 recruits EZH2 to chromatin [183]. YY1 mutants with decreased dimerization propensity show stronger binding to EZH2 [113], suggesting that dimerization might compete with EZH2 binding. Thus, the following mechanism can be proposed: zinc concentration in prostate cells decreases upon transformation, causing YY1 to monomerize. This enables YY1 binding to EZH2 and recruiting of the latter to chromatin. Histone methylation decreases expression of tumor suppressors, enabling further uncontrolled proliferation. 

### 7.2. AR

YY1 was shown by Deng and colleagues to interact directly with AR and regulate its transcriptional activity in a concentration-dependent manner in prostate cancer cells [142]. Decreasing YY1 concentration through silencing was observed to lower the expression level of genes controlled by AR. However, YY1 overexpression would also decrease the expression level of AR-targeted genes. To explain these seemingly contradictory observations, Deng at al. proposed a “squelching effect” mechanism, in which abundant YY1 molecules interact individually with particular cofactors required to form an effective transcriptional complex. It is worth noting that the “squelching effect” could result not only from increased YY1 concentration, but also from its diminished propensity for dimerization. In high zinc concentration, YY1 dimers could effectively bridge AR with its required cofactors. Decreasing zinc level, e.g., during propagation of prostate cancer, would destabilize the AR’s transcriptional complexes. 

### 7.3. MDM2/p53

YY1 interacts with p53 and with its ubiquitin ligase MDM2 [5]. Ubiquitination of p53 with MDM2 leads to its proteasomal degradation and thus can promote tumor progression by avoiding apoptosis [257]. Sui and colleagues showed through coimmunoprecipitation that YY1’s region 205–299 (equivalent to IR4 and 5) is necessary for physical interaction with MDM2. Interestingly, their genetic rescue experiments indicated diminished rescue ability of YY1 upon deletion of the 205–299 region, but also upon deletion of the histidine cluster (65–80) [5]. This suggests that the YY1 species involved in the interaction with MDM2 and p53 is actually the YY1 dimer, with one protomer binding to MDM2 and the other binding to p53. Since YY1’s histidine cluster is needed for its dimerization [9], its deletion would hamper the YY1-induced colocalization of MDM2 and p53, decreasing p53 ubiquitination by MDM. Indeed, YY1 dimerization promotes its interaction with MDM2, as shown by Qiao and colleagues [113] for YY1 mutants with decreased dimerization propensity.

### 7.4. YY1 Dimers in Chromatin Loops

Several previous examples highlight the role of YY1 dimerization in particular tertiary complexes. However, there is growing evidence that YY1 dimers take part in genome-wide transcriptional regulation by structuring the enhancer–promoter loops [6]. YY1 coordinates the formation of enhancer and super-enhancer complexes through liquid–liquid phase separation (LLPS) [258]. YY1’s histidine cluster is required for both LLPS [258] and for dimerization triggered by zinc ions [9]. This suggests that zinc ions might also promote LLPS of YY1, similarly to what was shown previously for other proteins, including tau [259,260], SOD-1 [261], or CTTNBP-2 [262]. Abnormal looping of genetic regulatory elements was observed in patients with Gabriele-de Vries syndrome, caused by mutations in the *yy1* gene [8], but can also happen in cells with the wild-type *yy1* in conditions that affect its dimerization propensity. For instance, prostate cancer cells are characterized by a decreased zinc level compared to healthy prostate, and they also exhibit abnormal architecture of higher-order chromatin elements, such as the transcriptionally associated domains, and dysregulated transcription profiles [263], in line with YY1 malfunction. 

### 7.5. E1A

Apart from the effect on prostate cancer oncogenes, zinc has been also recognized to be necessary for YY1 binding to adenoviral protein E1A [180]. E1A activates transcription by relieving YY1 repression. As a consequence, quiescent cells are induced to enter the cell cycle. The interaction engages two parts of YY1: 54–260 (equivalent to IR2-4) and 332-414 (IR7). The latter is composed of zinc finger motifs, which could explain the requirement for zinc. However, YY1’s IR2-4 also cannot bind E1A in the absence of zinc ions [180]. This suggests that their interaction requires the YY1 dimer. 

The proposed mechanism provides a possible explanation for YY1 malfunction in prostate cancer. Additionally, it implies potential therapeutic solutions aimed at restoring YY1’s dimeric structure. One possibility would be to increase the cellular zinc level. Zinc ionophores have proven to be promising agents in the treatment of prostate cancer [95,264]. Apart from the well-studied effect of zinc on cell metabolism and mitochondrial stability [105] or on downregulation of AR [265], an increase in zinc could also facilitate YY1 dimerization and thus disrupt its interactions with EZH2, AR, or MDM/p53. 

Contrary to prostate cancer, breast cancer is characterized by an increased cellular zinc level, overly promoting YY1 dimerization and oligomerization. In this case, decreased dimerization could be targeted with the use of peptides equivalent to YY1 fragments responsible for its dimerization. A peptide of the sequence of YY1’s OPB domain (IR4) was previously shown to inhibit YY1 binding to EZH2, reduce breast cancer cell viability, and efficiently inhibit the growth of a xenograft tumor [184,266]. Possibly, the zinc-chelating regions of YY1 could also be efficient inhibitors of anomalous YY1 activity resulting from zinc-driven oligomerization. 

## 8. Conclusions

The YY1 protein, with its intrinsically disordered regions, is highly likely to mediate interactions with multiple proteins, functioning in a partner-dependent manner within specific complexes. In proteins of this kind, any modification to the structural properties of the region involved in the interaction with a molecular partner can impact their mutual affinities, consequently promoting or inhibiting the formation of transcription factor complexes crucial for regulating the expression of specific genes. The existence of a diverse array of protein components within these transcriptional complexes leads to the observation that structural modifications can yield various, even opposing, effects on the activity of these complexes. As a result, a factor that modifies the structural changes can either enhance or diminish the transcriptional activity of the complex, depending on the involvement of the modified protein region in the essential interactions for that specific complex.

The recently demonstrated ability of YY1 to specifically bind zinc ions leads to changes that can significantly impact the protein’s activity. These changes must be considered in the context of specific transcriptional complexes, as the presence of zinc ions can modulate their formation in diverse ways. In consequence, changes in zinc levels may affect YY1’s activity in various ways, leading to alterations in gene expression patterns and potentially resulting in neoplastic transformation or tumor progression. As demonstrated by our examples, zinc ions can play a previously unnoticed and crucial role. This role may be particularly relevant in the process of carcinogenesis, where disease advancement is often correlated with alterations in the natural zinc concentration within a given tissue. The observed dependence of YY1’s structure and activity on the zinc level suggests previously unrecognized potential therapeutic applications. 

## Figures and Tables

**Table 1:** Proteins involved in the regulation of zinc levels in cells and their compartments.

Family	Protein	Flux	Expression	Reference
**ZIP**	ZIP1	EC → Cyt ER → Cyt	Ubiquitously expressed. In enterocytes and prostate epithelium located in basolateral membrane. Downregulated in prostate cancer and clear cell renal cell carcinoma. Expression inversely correlates with prognosis; enhances proliferation and invasion of ACHN cells. Upregulated in pancreatic cancer.	[12,13,14,15,16,17]
	ZIP2	EC → Cyt	Expressed in the apical membrane of prostate and uterine epithelial cells. Downregulated in prostate cancer	[15,18]
	ZIP3	EC → Cyt	Expressed in ovary, testis, pancreas, mammary gland and in the apical membrane of prostate epithelium. Takes part in zinc reuptake in the lactating mammary gland. Downregulated in prostate cancer and pancreatic cancer. Upregulated in pancreatic cancer	[15,17,19,20,21]
	ZIP4	EC → Cyt	Expressed in kidney, stomach, intestine, and colon. In enterocytes, located in apical membrane. Overexpressed in pancreatic, ovarian cancer and pancreatic cancer. Promotes proliferation of pancreatic cancer cells. Promoting invasiveness of hepatocellular carcinoma, nasopharyngeal carcinoma and non-small cell lung cancer. Overexpression predicts poor survival in acute myeloid leukemia and pancreatic cancer. Associated with cisplatin resistance, radioresistance and enhanced cell migration. Mutations cause acrodermatitis enteropathica	[17,22,23,24,25,26,27,28,29,30]
	ZIP5	EC → Cyt	Expressed in liver, kidney, pancreas, small intestine, colon, spleen. In enterocytes, located in basolateral membrane. Downregulated in pancreatic cancer	[17,31]
	ZIP6	EC → Cyt	Expressed in breast, prostate, placenta, kidney, pituitary gland. Triggers progression of breast cancer. Overexpressed in esophageal carcinoma and pancreatic and colorectal cancer. Overexpression promotes epithelial-to-mesenchymal transition, cell mobility and metastasis. Involved in antigen presentation to T cells	[17,32,33,34,35]
	ZIP7	ER → Cyt Golgi → Cyt	Ubiquitously expressed. Overexpressed and involved in progression of cancers of breast and cervix. Upregulated in pancreatic and colorectal cancer. Overexpression promotes cell proliferation, migration and invasion in breast and cervix cancer	[17,36,37,38,39]
	ZIP8	EC → Cyt	Ubiquitously expressed. Involved in inflammation.	[40,41]
	ZIP9	EC → Cyt Golgi → Cyt M → Cyt	Highest expression in pancreas, testis and pituitary gland. Upregulated in pancreatic and colorectal cancer. Acts as membrane androgen receptor. Involved in testosterone-induced apoptosis of breast and prostate cancer cells	[17,42,43,44,45,46]
	ZIP10	EC → Cyt	Highest expression in kidney. Involved in anti-apoptotic signaling during B cell development. Overexpressed and involved in progression of metastatic breast cancer and renal cell carcinoma. Upregulated in pancreatic and colorectal cancer. Promotes cell migration	[17,47,48,49,50]
	ZIP11	Golgi → Cyt N → Cyt	Expressed in testes, stomach, intestine; Involved in progression of pancreatic, ovarian, and cervical cancer. Variants associated with increased risks of bladder cancer, and renal cell carcinoma. Promotes proliferation of Capan-1 cells. Promotes proliferation, migration, invasiveness and mitochondrial potential of HeLa cells. Overexpression correlates with poor prognosis in cervical cancer	[17,51,52,53]
	ZIP12	EC → Cyt	Highest expression in brain, eye and pulmonary vasculature.	[54,55,56]
	ZIP13	ER → Cyt Golgi → Cyt	Highest expression in heart, dermis and connective tissue. Upregulated in pancreatic cancer	[17,57,58,59,60]
	ZIP14	EC → Cyt M → Cyt	Zinc uptake to liver in response to inflammation. Regulates glucose homeostasis in hepatocytes. Overexpressed in hepatocellular carcinoma. Downregulated in pancreatic cancer	[17,27,54,61,62]
**ZnT**	ZnT1	Cyt. → N Cyt. → EC	In enterocytes, located in basolateral membrane. Upregulated in pancreatic cancer. Promotes proliferation of Capan-1 cells. Underexpression predicts poor survival in acute myeloid leukemia	[17,24]
	ZnT2	Cyt. → ER Cyt. → E	Overexpressed in breast cancer. Serves for vesicular sequestration of the accumulated zinc. Protecting the cells from zinc cytotoxicity. Downregulated in pancreatic cancer	[17,63]
	ZnT3	Cyt. → SV Cyt. → E	Transports zinc into synaptic vesicles of glutamatergic neurons	[64]
	ZnT4	Cyt. → Golgi Cyt. → SV Cyt. → E	Higher expression in prostate than in other organs. Expression decreases upon the progression of prostate cancer. Overexpressed in liver and pancreas cancer and glioma. Repressing apoptosis. Enhancing proliferation and migration. Higher expression predicted poorer survival in acute myeloid leukemia	[23,30,65,66,67]
	ZnT5	Cyt. → Golgi Cyt. → IG	Highly expressed in pancreas, liver, kidney and insulin-containing beta cells. Undetectable in other endocrine cell types. Decreased expression predicts poor survival in acute myeloid leukemia. Expression is regulated by zinc ions. Upregulated by endoplasmic reticulum stress. Lower expression predicted poorer survival in acute myeloid leukemia. Upregulated in pancreatic and colorectal cancers	[17,30,68,69,70,71,72,73,74]
	ZnT6	Cyt. → ER Cyt. → Golgi	Expressed in brain, B-cells, colon, eye, and lung. Lower expression in bone, brain, cervix, ear, heart, kidney, muscle, nerve, pancreas, prostate, skin, stomach, and testis. Upregulated in pancreatic and colorectal cancers. Promotes proliferation of Capan-1 cells. Underexpression predicts poor survival in acute myeloid leukemia	[17,74,75,76]
	ZnT7	Cyt. → ER Cyt. → Golgi	Upregulated in pancreatic and colorectal cancers. Lower expression predicted poorer survival in acute myeloid leukemia	[17,30,74]
	ZnT8	Cyt. → IG	Expressed in pancreatic beta-cell-specific zinc transporter. Precent in fat tissue and T-cells and B-cells	[77,78,79]
	ZnT9	Cyt. → N	Ubiquitously expressed in fetal and adult tissues and cancer cell lines. Mitochondrial zinc exporter. Upregulated in pancreatic cancer	[17,80,81]
	ZnT10	Cyt. → Golgi Cyt. → E Cyt. → EC	Highest levels of expression in small intestine, liver and brain tissues. Down-regulated by zinc and angiotensin-2	[82,83,84]
**MT**	MT1	Cytoplasm sequestration	Ubiquitously expressed. Overexpressed in cancers of the breast, colon, kidney, liver, skin (melanoma), lung, nasopharynx, ovary, prostate, mouth, salivary gland, testes, thyroid and urinary bladder. Underexpressed in hepatocellular carcinoma, liver adenocarcinoma and pancreatic cancer	[17,85]
	MT2a		Ubiquitously expressed. Overexpression correlates with cancer chemoresistance and acute myeloid leukemia	[86,87]
	MT3		Expressed mainly in central nervous system. Present in kidney and jejunum. Abundant in a subset of astrocytes in the normal human brain	[88,89]
	MT4		Specifically expressed in stratified epithelia	[90,91]

The following abbreviation have been used: ER—endoplasmic reticulum; Cyt.—cytoplasm; EC—extracellular; N—nucleus, M—mitochondria; E—endosome; SV—synaptic vesicle; IG—insulin granule.
